# First person – Katja Graf and Antonia Last

**DOI:** 10.1242/dmm.041970

**Published:** 2019-09-01

## Abstract

First Person is a series of interviews with the first authors of a selection of papers published in Disease Models & Mechanisms (DMM), helping early-career researchers promote themselves alongside their papers. Katja Graf and Antonia Last are co-first authors on ‘ Keeping *Candida* commensal: how lactobacilli antagonize pathogenicity of *Candida albicans* in an *in vitro* gut model’, published in DMM. Katja is a postdoc and Antonia is a PhD student in the lab of Bernhard Hube at the Hans Knöll Institute (HKI) investigating molecular pathogenicity mechanisms.


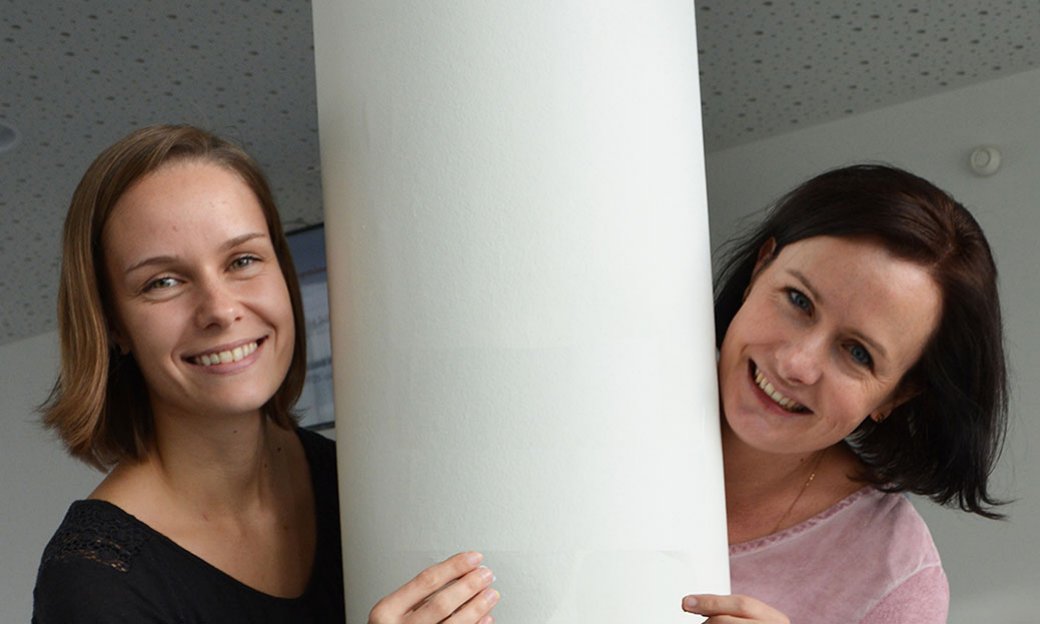


**Antonia Last and Katja Graf**

**How would you explain the main findings of your paper to non-scientific family and friends?**

AL+KG: We work with the fungus *Candida albicans*, which is better known as the cause of vaginal or oral yeast infections (thrush). These infections affect millions of individuals all over the world. However, this fungus is found in most people on mucosal surfaces, including the human gut, living in harmony with harmless and beneficial gut bacteria, together forming a complex community of microorganisms – the microbiota. Under certain circumstances this balanced microbiota can be disrupted, for example by the use of broad-spectrum antibiotics, which remove most bacteria, both beneficial and disease-causing, but not the fungi. This allows *C. albicans* to overgrow everything else and potentially trigger an infection (candidiasis). In rare worst cases, this fungus can enter the bloodstream, causing systemic infections and sepsis with an often fatal outcome. Lactobacilli are well-known protectors of mucosal barriers that help to maintain microbial balance, but the reasons for that are not fully understood. In our model, we proved that *Lactobacillus rhamnosus* protects gut barriers against *C. albicans* damage and obtained interesting insights into the mechanism. When both *C. albicans* and lactobacilli are present, some of the epithelial cells lining the gut commit a form of ‘cellular suicide’ (apoptosis). These dead human cells, the fungal cells and the bacteria clump together and are thereby removed from the remaining healthy human cells. We assume that this physical separation can protect the gut barrier from invasion by the fungus and prevent a disease at the earliest stage.

**What are the potential implications of these results for your field of research?**

AL+KG: We established an *in vitro* model based on intestinal epithelial cells and mucus-secreting goblet cells. In this model, we added an artificial microbiota in the form of lactobacilli to mimic the *in vivo* situation. This more complex model is closer to reality than many other models and a good basis for all kinds of research into interactions of gut cells with commensal or pathogenic members of the microbiota. Our study shows an unexpected interaction between the intestinal epithelial cells, probiotic bacteria and the opportunistic fungal pathogen *C. albicans*, which relies on physical separation induced by the presence of the bacteria. This model and its read-out can now be used to test other bacteria or antifungals for their protective potential against *Candida-*induced damage. Even pathogenic bacteria could be tested, to model polymicrobial infections. Given the renewed interest in probiotics and the role of the microbiota in health, such easily accessible *in vitro* models are, in our opinion, urgently needed.

**What are the main advantages and drawbacks of the model system you have used as it relates to the disease you are investigating?**

AL: One big advantage of our *in vitro* model is the fact that we are working *in vitro*, which is much easier to plan and to work with compared to *in vivo* models, and is generally more reproducible and ethically preferred. Personally, I really enjoyed the fact that the cell culture cells had to differentiate for two weeks before we could use them, as that gave me enough time to prepare my experiments thoroughly and re-think everything before conducting the experiment. On the other hand, it is extremely frustrating and time consuming if something goes wrong; a contamination in your cell culture can delay your next experiment by three to four weeks! All in all, it was a good exercise for me to plan experiments in advance.

“Working *in vitro* is much easier to plan and to work with compared to *in vivo* models, and is generally more reproducible and ethically preferred.” – *Antonia Last*

KG: Our *in vitro* model is more complex than most established cell line-based models, which usually consist of only one host cell type. We included two different gut cell types and additional probiotic bacteria to come closer to the real *in vivo* situation. This gives us the chance to study the complex cell-bacteria-fungal interactions in a more life-like manner. However, one important part is still missing in our model: immune cells, which also play a major role during commensalism and pathogenicity in the gut. We plan to include these cell types, although it will make it more complicated to work with. As is so often the case, we could lose the benefits of our reductionist approach by adding complexity. At the end, every *in vitro* model is, to varying degrees, artificial, and we are happy that we were able to establish this more complex model to study fungal infection biology.

**Figure:**
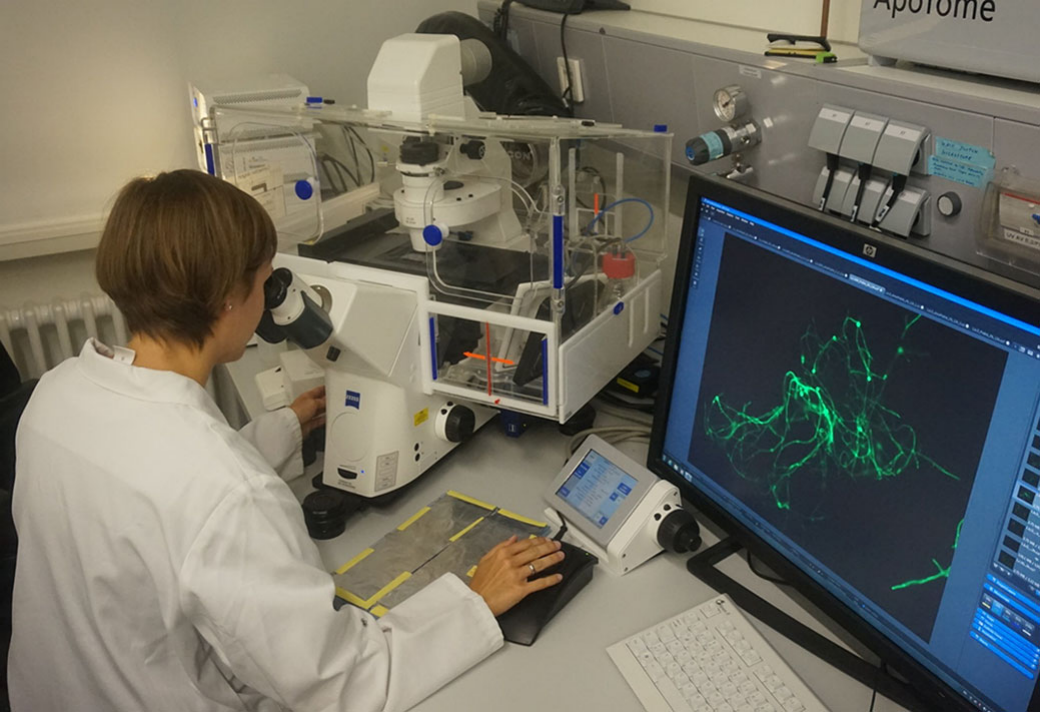
**Antonia Last studying *C. albicans* at a fluorescent microscope.**

**What has surprised you the most while conducting your research?**

KG: In our publication we explain that the protective effect of lactobacilli against *C. albicans*-induced damage is due to shedding of fungal, bacterial and apoptotic host cells. We saw this shedding by eye at the beginning of our studies, but were not really considering it as relevant. It was only when we saw aggregation of the microbes and human cells via electron microscopy, that we gave this original observation a second thought and based a new hypotheses on it. I am frequently surprised that such seemingly irrelevant observations can turn out to be the most important hints in science, so I can only recommend keeping your eyes open!

AL: I was extremely surprised how many new and useful results you can get during the revision process after submission! It was quite a lot of work for us, but through the reviewers’ helpful comments and suggestions, we were able to improve our publication significantly. This is probably one of the more underestimated factors in science.

“Seemingly irrelevant observations can turn out to be the most important hints in science, so I can only recommend keeping your eyes open!” – *Katya Graf*

**Describe what you think is the most significant challenge impacting your research at this time and how will this be addressed over the next 10 years?**

KG: The number of (nosocomial) *C. albicans* infections and related sepsis cases is increasing. This puts fungal infections more in focus nowadays. Even though *C. albicans* research is already comparatively well established (for a fungal pathogen), many pathogenicity mechanisms of the fungus remain unknown. Its interaction with the human host, and especially its complex interplay with the microbiota (an increasingly recognized contributor to health and disease), requires much more research. The main challenge here is probably the ‘black box’ gut, as we still don't have a complete picture of the resident microbiota, and especially not of the myriad of interactions within this unique biosphere. If we want to prevent and cure life-threatening *C. albicans* infections, we need to know much more about the natural habitat of the fungus, its neighbours and the human gut in general.

**What changes do you think could improve the professional lives of early-career scientists?**

AL: From my point of view, it is very helpful to be part of a graduate school as a PhD student. I have the opportunity to join soft-skill courses, scientific courses and seminars as well as national and international conferences. All these experiences help me to get a broader view into the life of a researcher and allow me to improve the skills necessary for this kind of work. Moreover, collaborations can be very fruitful, as they give new input and broaden the spectrum of equipment and expertise. But next to all these, the most important thing to have for a successful PhD is the supervision. I am really thankful that in our department every PhD student has an experienced postdoc on their side. To have someone to discuss problems and ideas with is not only extremely useful, but absolutely necessary in this phase of the career.

**What's next for you?**

AL+KG: We would like to get deeper insights into the described protective mechanism of lactobacilli against *C. albicans*-induced damage. In order to further increase the biological complexity of our model we have teamed up with the research group of Alexander Mosig at the University Hospital in Jena and are working on establishing our model in their intestine-on-chip model (Maurer et al., 2019). In addition, we are currently conducting experiments in our *in vitro* set up, in which we perform transcriptional and metabolic profiling. We hope the new results will answer some questions about the gene regulation involved during the interactions between lactobacilli, *C. albicans* and intestinal epithelial cells. Another aim is the introduction of immune cells in our model, to investigate the contribution of another important player in the gut.
